# The Evidence Regarding Maintenance Tocolysis

**DOI:** 10.1155/2013/708023

**Published:** 2013-03-14

**Authors:** John P. Elliott, John C. Morrison

**Affiliations:** ^1^Perinatal Services, Division of Southwest Contemporary Women's Care, Southwest Perinatal Services, 16611 S. 40th Street, Phoenix, AZ 85048, USA; ^2^Department of OB/GYN, University of Mississippi Medical Center, Jackson, MS 39216, USA

## Abstract

Preterm delivery is a public health issue of major proportion. More than 12% of deliveries in the United States that occur at less than 37 weeks gestation preterm labor (PTL) represents the largest single reason for preterm birth (PTB). Attempts to prevent PTB have been unsuccessful. This paper of maintenance tocolytic therapy will examine the efficacy and safety of the drugs, both oral and subcutaneous, which have been utilized for prolongation of pregnancy following successful arrest of a documented episode of acute preterm labor. The evidence for oral tocolytics as maintenance therapy as well as parenteral medications for such patients is offered. Finally, the effects in the United States of the Food and Drug Administration (FDA) action on such medications are reported.

## 1. Introduction

The rate of preterm birth (PTB) defined as less than 37 weeks gestation is unacceptably high worldwide. In the US, it exceeds 12% [1]. PTB is responsible for up to 75% of infant mortality and up to 50% of long-term neurologic deficits [2]. The majority of these deliveries result from either preterm labor or preterm premature rupture of the membranes accompanied by preterm labor [3]. Acute preterm labor at 20 0/7 to 36 6/7 weeks can be successfully treated in 70–90% of patients if the diagnosis is made prior to advanced cervical dilation (≥3 cm) [4]. This success rate is not achieved if there is chorioamnionitis or placental abruption causing PTL. However, once these women are sent home following successful tocolysis, recurrent preterm labor and/or preterm premature rupture of the membranes is likely, and these patients are at high risk to deliver at <37 weeks' gestation [5]. There are several choices of drugs for maintenance tocolysis after acute preterm labor is arrested, but some feel that treatment does not significantly prolong gestation [6]. For example, oral tocolysis with beta-agonist or with oral magnesium has been shown in randomized clinical trials not to extend gestation or benefit the neonate [5, 7]. Similarly oral nifedipine maintenance tocolysis has not been shown to be beneficial [8]. However, failure of maintenance tocolysis may have more to do with the agent and its side effects (thus compliance) than with tocolytic drug efficacy. There is also an effect of the route of delivery of the tocolytic drug on efficacy and compliance. For example, the oxytocin receptor antagonist Atosiban given parenterally and the continuous subcutaneous administration of terbutaline supplemented by daily perinatal nursing calls and home uterine activity monitoring, both have been shown to prolong the interval from treatment to delivery and result in less preterm birth, while improving composite neonatal morbidity [9, 10].

The purpose of this study is to present the evidence regarding maintenance tocolysis in women following arrest of confirmed preterm labor episode who are discharged from the hospital.

## 2. Oral Maintenance Therapy

Once confirmed preterm labor has been stopped by parenteral or oral tocolytic therapy, the patients are usually discharged on no treatment, oral therapy using magnesium, beta-agonists, or a calcium channel blocker, or maintenance drugs such as terbutaline or an oxytocin antagonist administered subcutaneously. The Section 3 will deal with oral tocolytics. The results of maintenance therapy have been conflicting, as many trials could not document that the oral medications were taken effectively due to side effects versus other trials having no comparison group. Finally, adding to the difficulty of assessing the evidence is the endpoint: preventing preterm births at <37 weeks' gestation versus prolonging pregnancy.

### 2.1. Oral Beta-Agonist

Creasy et al. [57] first demonstrated that oral ritodrine extended gestation after arrest of acute preterm labor when compared to placebo [57]. Lewis et al. [58], in contrast, found that compared to placebo, oral terbutaline was not effective in prolonging gestation or improving neonatal outcome [58]. In a meta-analysis of four trials, where oral beta-agonist or oral magnesium compounds were compared to placebo in women with confirmed tocolysis of acute preterm labor, Macones et al. [59] found that there was no benefit in extending gestation to 37 weeks, although there was an average of 4 weeks delay from the tocolytic treatment to delivery [59]. Rust et al. [5] studied 248 patients who randomized to receive oral terbutaline, oral magnesium, or placebo. The rate of preterm delivery at <37 or <34 weeks was similar in the three treatment groups. Significantly, these investigators noted that compliance with oral medications was between 55.4% in the magnesium arm (usually due to diarrhea) versus 63.9% in those taking terbutaline (usually discontinued for palpitations and/or nervousness). Similarly Parilla et al. [60] demonstrated that oral terbutaline given at home after successful intravenous magnesium sulfate did not reduce the rate of preterm birth.

In contrast, Brown and Tejani [61] reported a randomized trial of oral terbutaline (5 mg q 6 hours) for maintenance treatment and found a significantly longer treatment to delivery interval when the beta-agonist was compared to placebo. Also, infants born to mothers receiving oral terbutaline had a reduction in respiratory distress syndrome. Similarly, Caritis et al. [62] noted that terbutaline (30 mg/d) was more successful in prolonging pregnancy to 37 weeks when compared to ritodrine (120 mg/d). Also in that study, pregnancy prolongation was greater (40 days) with terbutaline when compared to ritodrine (22 days). Likewise, Berg et al. [63] gave oral maintenance terbutaline (5 mg tid) to women and found that the majority of patients had prolongation of pregnancy more than 7 days. More recently, Plummer [64] prospectively randomized 162 patients with a diagnosis of acute PTL into 3 groups: (1) no acute tocolysis; no maintenance tocolysis, (2) acute tocolysis; no maintenance tocolysis, and (3) acute tocolysis; oral maintenance therapy. The results showed a significant prolongation of pregnancy in patients in group 3 with maintenance therapy (*P* = .0001) but due to the small number of patients, there was no difference in maternal or neonatal outcomes.

### 2.2. Oral Magnesium

Martin et al. [65] studied two groups of patients who had been effectively tocolyzed after an acute episode of preterm labor. He noted that magnesium gluconate (2 gm/day) was more effective than placebo in lengthening the interval to delivery reducing the incidence of recurrent preterm labor. However, there were not enough patients to show statistical significance. Oral magnesium was also used as prophylaxis in patients who were at high risk for preterm labor and who had low magnesium levels [66]. There was no difference in magnesium gluconate and placebo with respect to the incidence of preterm labor or the time to delivery in those who developed early labor and were tocolyzed. The trial by Rust et al. [5] also included a magnesium chloride arm for oral tocolysis. The number of deliveries before 37 weeks was not different from the placebo group, nor was birth weight or neonatal ICU admission.

### 2.3. Oral Calcium Channel Antagonists

Oral or sublingual nifedipine administration for treatment of acute preterm labor has been the subject of many small nonrandomized prospective trials and meta-analysis [67]. More recently, nifedipine was studied for maintenance tocolysis in randomized clinical trials which have demonstrated no difference in outcome between the study drug and the control group [8, 68, 69]. El-Sayed et al. [68] in a small study randomized 69 women to maintenance therapy with oral diltiazem or oral nifedipine after preterm labor had been arrested. There were no significant differences in interval from treatment o delivery, gestational age at delivery, or adverse effects. Similarly, Lyell et al. [8]showed that when compared to placebo maintenance tocolysis, nifedipine did not reduce preterm birth, prolong pregnancy, or improve neonatal outcome [8]. Finally, Carr et al. [69] randomized 74 women between 24 and 34 weeks' gestation to receive maintenance tocolysis with oral nifedipine (20 mg every 4–6 hours, *n* = 37) or no treatment (control, *n* = 37) after acute preterm labor was arrested. While all factors were balanced between the two groups and both were randomly assigned to receive drug or no drug, the time gained in pregnancy with nifedipine (37 ± 23.1 days versus 32.8 ± 20.4 days) in the control group was not different (*P* = .44). Similar findings were noted when oral nifedipine was matched to placebo for gestational age at delivery (35.4 ± 3.2 weeks versus 35.3 ± 3.2 weeks). Moreover the incidence of side effects, with nifedipine includes hypotension, tachycardia, pulmonary edema, and even fetal death in case reports or small series [70–72]. Similarly, when magnesium is being given for preeclampsia, treatment for preterm labor with terbutaline can clinically be associated with significant hypocalcemia [73]. The authors conclude that maintenance therapy with oral nifedipine after arrest of acute preterm labor did not significantly prolong pregnancy or improve neonatal outcome.

### 2.4. Antiprostaglandin Drugs

While antiprostaglandin drugs, administered as suppositories or orally, have long been accepted as primary and secondary treatment for acute preterm labor, there is no data regarding the use of these drugs for maintenance therapy [67]. Even, when used at <32 weeks and <48 hours in a treatment cycle, the incidence of oligohydramnios and ductal constriction are too high to justify its use as maintenance treatment after arrest of acute preterm labor [74].

### 2.5. Summary of Oral Maintenance Tocolysis

The available literature on oral maintenance tocolysis is conflicting. There are obviously issues about dosing of oral drugs. Can a therapeutic level of drug be achieved without eliciting unacceptable side effects that prompt patients to be noncompliant [5]? In addition, the study design of all trials of oral maintenance tocolysis have compared arbitrarily; the dosage of drugs and interval of administration without consideration of confounding issues such as volume of distribution or excretion rate. 

The literature also has not directly addressed whether the drug actually had a biological effect on the frequency of contractions. This is important as the goal of maintenance therapy is to suppress contractions which then might prevent or postpone another episode of PTL. There are some studies that address this point tangentially. There are several studies that have reported what happens when the drugs are electively discontinued [43, 54]. Thirty to 50% of these patients deliver within 72 hours and 70–80% within 7 days. It is very unlikely that 70–80% of patients would deliver within 7 days of discontinuation of oral maintenance therapy if the drug was not providing suppression of contractions. Therefore, oral tocolytics when given in a maintenance form may not seem to work, but it may be that the effective dosage of medications have side effects resulting in poor compliance or discontinuation of treatment [5]. A meta-analysis by Meirowitz et al. [75] concluded that they could not resolve the issue of the effect of oral maintenance tocolysis because the trials were so different and the population so small in number. Therefore, many conclude that oral maintenance tocolytics have no value in continuing pregnancy and should not be used [6, 7]. While it is not possible to leave all patients in the hospital after an episode of acute preterm labor, if one is not going to use maintenance tocolysis on an ambulatory basis, at the very least, home contraction monitoring with daily nursing calls should be utilized in our opinion to detect recurrent preterm labor at the earliest time [10].

## 3. Subcutaneous Maintenance Tocolysis

### 3.1. Oxytocin Antagonists

Valenzuela et al. [9] studied the oxytocin receptor antagonist, Atosiban, administered via a subcutaneous infusion pump versus a placebo in a blinded, randomized control study in women with arrested preterm labor. Two hundred and sixty-one subjects were randomized to Atosiban while 251 received placebo parenterally. The primary endpoint was the interval from the start of therapy to the first recurrence of preterm labor. The interval was 33 days in the Atosiban group versus 27 days in those receiving placebo (*P* = .025). While there were less births <32 weeks and fewer deliveries at 48 hours in the Atosiban group, the number of patients delivering at <37 weeks was not statistically different. There were no significant differences in neonatal outcome between the two groups. The Food and Drug Administration (FDA) stated that Atosiban posed no safety risks compared to placebo but suggested, since there was no decrease in preterm birth, that the drug could not be approved. There have been six additional trials on Atosiban for treatment of acute preterm labor, but none studied maintenance tocolysis [76]. More recently, Husslein et al. [77] performed an open label randomized trial comparing Atosiban to other tocolytic agents. Two hundred and ninety-five women received Atosiban versus 290 who received other tocolytics. Similar to the American trial, more women remained undelivered in the Atosiban group at 48 hours (77.6% versus 56.6%, *P* = .001) and more women in the Atosiban treatment group required no additional tocolytics (85.1% versus 62.8%, *P* = .001) compared to no treatment or other tocolytics. Maternal and fetal safety was significantly improved with Atosiban, but neonatal outcome was the same. In summary, while maintenance oxytocin receptor drugs are used for maintenance tocolysis in other countries, it is not available for treatment in the United States because the drug was not approved by the FDA which stated that the studies available did not find a difference in births <37 weeks' gestation or neonatal outcome between the two groups [9].

## 4. Continuous Subcutaneous Terbutaline Infusion—Efficacy

First introduced in 1987, there are now 46 peer-reviewed published studies regarding the programmable subcutaneous infusion of terbutaline for treatment of preterm labor, and all are listed in Table 1. Forty-four of these studies include over 25,000 patients of which 21,595 received subcutaneous terbutaline infusion for maintenance tocolysis and showed benefit in prolongation of pregnancy (greater gestational age at delivery), as well as decreased neonatal complications and/or maternal-newborn cost reduction compared to other therapy or placebo. These studies are Level II, (26 observational case control/cohort studies) and III (17 descriptive case series) evidence with one Level I study by Lam et al. [11]. There are also two Level I studies on subcutaneous terbutaline infusion for maintenance tocolysis which found no difference between terbutaline pump infusion and placebo [29, 30]. There are major flaws with these two studies, which enrolled a total of 94 patients with only 39 receiving subcutaneous infusion of terbutaline for tocolysis. Although these studies did not show a positive benefit for terbutaline for tocolysis, the investigators did not evaluate the therapy as it is used in clinical practice. For example, the bolus drug dosage administered in these two studies was not altered based on end-organ response (uterine contraction) data, as is required if any benefit is to be achieved, because the research protocol did not allow uterine contraction monitoring. Another problem in the design of these two studies is that the investigators did not use pharmacologic consultation regarding volume of distribution, body mass, renal clearance, and so forth to determine the appropriate basal dose of terbutaline for infusion, but rather all patients (BMI 25 versus 45) received the same fixed dosage of the drug regardless of the BMI. For example, the Wenstrom et al. [29] study used only four subcutaneous boluses of .25 mg of terbutaline or placebo with no adjustment based on end-organ response (contractions). Success with tocolytic drugs has been shown to be dose dependent with higher doses of the drug, for example, magnesium sulfate, to improve success rates [4]. In clinical practice, using the terbutaline pump, eight or more bolus doses are often necessary for successful tocolysis; not four for every patient as in this study [10]. Both studies were also underpowered (94 enrolled, 320 required by their power calculation) to show a difference from placebo. The Guinn study [30] is also compromised by the high dropout rate 41% in both arms of the study, (11 out of 24 patients randomized to the terbutaline arm and 9 of 28 patients in the placebo arm). Despite an intention to treat analysis, there were only 13 patients in each arm of the study actually utilizing therapy. Indeed, a conclusion in the Wenstrom article [29] states: “We cannot exclude the possibility that a much larger study would reveal some differences in outcome.” Eliminating these two studies based on valid scientific methodological errors leaves only positive studies supporting the effectiveness of terbutaline pump tocolysis.

There are three publications [43, 54, 78] that present efficacy data concerning the time to delivery after discontinuation of maintenance tocolysis with terbutaline pump therapy. Morrison et al. [78] studied 69 patients with a documented diagnosis of PTL; 41 treated with oral betamimetic maintenance tocolysis and 28 with terbutaline pump therapy. These patients were stable, without excess contractions/cervical dilatation, when the tocolytic medication was discontinued at 37 weeks. Forty-one percent (29/69) of these patients delivered <24 hours after the medication was discontinued. This compared to 4/41 (10%) in a control group with similar risk factors who were treated by the attending physician with oral betamimetic prophylaxis (no episode of PTL in this pregnancy). Jones et al. [43] reported data on interval from elective discontinuation of terbutaline pump therapy at 33.0–35.9 weeks gestation. These patients were stable without excess uterine activity on terbutaline pump therapy and were not hospitalized at the time of discontinuation. Fourteen hundred twenty patients were studied. They found that 50% of singletons and 83% of twins delivered ≤7 days from discontinuation of therapy while 33% of singletons and 60% of twins delivered ≤3 days. The mean time from discontinuation to delivery was 9.8 ± 10.1 days. Rebarber et al. [54] published a study consisting of 4253 singleton gestations receiving terbutaline pump therapy who had the pump discontinued by the attending obstetrician electively between 33.0–36.9 weeks after many weeks of successful therapy. The median time from discontinuation of terbutaline pump therapy to delivery was 5 days. Overall, 2316/4253 (55%) delivered <37 weeks (preterm); 1752/4253 delivered ≤3 days; and 2472/4253 (58.1%) delivered ≤7 days after discontinuation of the drug. Delivery of ≥50% of patients ≤7 days from discontinuation of therapy is extraordinary since all these women had confirmed PTL and achieved uterine quiescence for an average of 6–10 weeks. The authors conclude that the terbutaline pump was responsible for the lack of recurrent preterm labor and possible early delivery. When one reviews all the efficacy data regarding subcutaneous terbutaline infusion for maintenance tocolysis, it appears that in appropriate patients, this therapy significantly prolongs pregnancy, which results in later delivery, less NICU admissions with fewer neonatal complications, thus reducing costs (Table 1, references).

### 4.1. Continuous Subcutaneous Terbutaline Infusion Safety

To obtain a clear picture of the evidence on safety, one must return to the era of intravenous infusion of beta-agonists such as Ritodrine and terbutaline. The drug Ritodrine received FDA approval for intravenous treatment of PTL in 1977. This drug was eventually removed from the United States market by the manufacturer because of cardiovascular complications including maternal death in 24 patients. Those deaths were primarily due to hyperkalemia causing fatal arrhythmias. Terbutaline was also administered intravenously to treat acute preterm labor, and it had the same beta-agonist side effects as Ritodrine and thus obstetricians in the United States abandoned intravenous terbutaline and switched to magnesium sulfate (MgSo_4_) or other treatment for acute preterm labor. Terbutaline administration by a subcutaneous programmable pump was first advocated by Lam et al. in 1987 and tens of thousands of pregnant women each year use this method of treatment. In 1997, the FDA issued a Dear Colleague letter “to notify healthcare professionals about concerns regarding the safety of long-term subcutaneous administration of terbutaline.” The precaution section of the labeling was revised to warn about serious adverse reactions, including adverse cardiovascular events that may occur after administration of terbutaline to women in labor. The data appears to refer to intravenous terbutaline for acute PTL not subcutaneous maintenance therapy. The use of continuous subcutaneous therapy continued, but on February 17, 2011, the FDA issued “FDA Drug Safety Communication: New warnings against use of terbutaline to treat preterm labor.” In that communication “The US Food and Drug Administration (FDA) is warning the public that INJECTABLE terbutaline should not be used in pregnant women for prevention or prolonged treatment (beyond 48–72 hours) of preterm labor in either the hospital or outpatient setting because of the potential for serious maternal heart problems and death. The agency is requiring the addition of a BOXED WARNING and CONTRAINDICATION to the terbutaline tablet label to warn against its use.”

It is important to understand the basis for the FDA action regarding the use of subcutaneous maintenance terbutaline, which ultimately resulted in the manufacturer voluntarily removing the drug from the market. The FDA reviewed postmarketing reports of maternal deaths and serious cardiovascular adverse events submitted to the Adverse Event Reporting System (AERS). There were 16 maternal deaths reported since the initial marketing of the drug in 1976 until 2009 (33 years). In that time, it is estimated that terbutaline has been used in 250,000 pregnancies per year (mostly subcutaneous and oral administration since 1985) in the United States (8,250,000 pregnant women). This would be a mortality rate of 0.19/100,000. The maternal mortality rate (MMR) overall in the US is approximately 12.1/100,000 in 2003 (PRN) [4]. Thus, the reported mortality in women taking terbutaline appears to be much lower than the general mortality rate in pregnancy. Of the 16 maternal deaths, 3 were in patients using home terbutaline pumps 9 involved oral terbutaline (no subcutaneous terbutaline) or intravenous terbutaline. The remaining 4 deaths were “subcutaneous, intravenous or unknown.” Information about these 16 deaths, specifically any comorbidities such as underlying cardiac problems, concurrent medical problems, or the cause of death, has not been released by the FDA. Two of the deaths occurred in nonhospitalized patients on terbutaline pump tocolysis and they have been further studied. Perry et al. [27] reported 2 maternal deaths in a study population of 24,406 patients over 6 years (1987–1993). Both deaths were not related to terbutaline (1 ruptured iliac aneurysm and 1 patient with twins in whom the autopsy proven cause of death was hypoglycemia rather than hyperglycemia, which is related to beta-agonist therapy). Elliott et al. [39] presented further data in 9359 patients receiving subcutaneous terbutaline therapy covering 1995–2000. There were no deaths in these patients. These two studies report no terbutaline caused maternal deaths in 33,765 patients over 12 years. Therefore, 9 of the 16 FDA AERS reported deaths did not involve subcutaneous administration, while 4 are unknown. Two of the 3 deaths have been studied in detail and did not appear to be related to terbutaline infusion.

The FDA AERS also reported 12 cases of “serious cardiovascular events associated with use of terbutaline” reported over nearly a decade between January 1, 1998 (after the dear colleague letter) and July 2009. The morbidities reported include cardiac arrhythmia, myocardial infarction, pulmonary edema, hypertension, and tachycardia. Only three of the 12 cases involved terbutaline pump maintenance therapy and 5 involved oral terbutaline ± subcutaneous injections. The other 4 cases appeared to involve intravenous terbutaline. Perry et al. [27] and Elliott et al. [39] reported .54% and .13% incidences of significant cardiovascular events in patients on terbutaline pump tocolysis. In both reports, cardiopulmonary complications almost always occurred during hospitalization, with concomitant tocolysis and/or in women with comorbidities such as multiple gestation or hypertension, that is, hypervolemic states. The majority of patients with these cardiovascular side effects were not even on the terbutaline pump maintenance therapy, when symptoms occurred and the cardiovascular problems diagnosed.

In addition to the Black Box warning, the FDA changed the risk category of terbutaline from B (no human or animal-reproduction studies have demonstrated a fetal risk) to category C (animal reproduction studies have shown an adverse effect on the fetus). The FDA referenced 16 studies which were the basis of the change and 14 of the references are data from one laboratory [79] (Slotkin). A recent publication by Owens et al. [80] attempted to replicate the harmful effects of terbutaline in rat pups published by Slotkin et al. [79, 81, 82]. The results of the Owens paper confirm that the type of terbutaline used in Slotkin's work was probably causative of the adverse effects noted in the fetal rat pups. Slotkin used pharmacologic grade terbutaline (Sigma Chemical Corp.) not medical grade (used in humans). Owens confirmed Slotkin's findings with pharmacologic grade drug but did not find any harmful effects when medicinal grade terbutaline was used even though the dosage used in animals was approximately 260 times the dose used clinically in pregnant women. It is unfortunate that the FDA used animal data from only one laboratory that was not replicated in other research studies (and refuted by the most current study [79]), to change the classification from B to C for terbutaline.

### 4.2. Continuous Subcutaneous Terbutaline Infusion—Summary

It is unfortunate that the decisions by the FDA to issue a Black Box Warning about the use of terbutaline sulfate in pregnancy and to change its classification from category B to C did not appear to be based on assessment of the vast majority of available evidence. Terbutaline pump tocolysis has been shown in 44 of the 46 peer-reviewed studies to significantly prolong gestation, reduce the incidence of recurrent preterm labor, which resulted in fewer NICU admissions and neonatal complications, and thus reduced the cost of medical care (Table 1, references). There are few documented cases of mortality and morbidity associated with terbutaline pump therapy, and most are associated with comorbidity and hospitalization after discontinuation of in-home terbutaline therapy. Actually, the safety profile of terbutaline using continuous subcutaneous terbutaline via infusion pump is actually quite good [27, 39]. In our opinion perhaps, the FDA should reconsider its decision because the harm to babies and their families due to PTD is a far greater public health impact than the small theoretical risk from terbutaline given in this fashion to pregnant women.

## 5. Conclusion

Maintenance tocolysis with orally administered drugs including nifidepine, magnesium, and terbutaline may be effective in delaying preterm birth in women having an episode of acute PTL which has been successfully treated. The efficacy is balanced against side effects that affect compliance and limit the ability to achieve therapeutic serum levels of these drugs. Because the evidence is conflicting, clinicians may choose to use an oral tocolytic or to follow the patient on no medication after discharge provided that they use some system to detect recurrent preterm labor at the earliest time. Further research should be directed toward documentation of a decrease in contractions after giving oral tocolytics to help determine the appropriate dose in that patient and the interval between doses that will allow prolongation of pregnancy with the least side effects.

Subcutaneous administration of Atosiban or terbutaline by infusion pump appears to be beneficial as maintenance tocolysis based on all the available evidence. It is very unfortunate that the action by the FDA has prevented the use of this technology in the United States, although it remains available in the rest of the world. The available evidence supports the efficacy and safety of subcutaneous terbutaline administered by a programmable infusion pump (see Table 1 with references). Finally, it should be remembered that maintenance tocolysis is only one part of preventing preterm deliveries. Screening patients for risk factors, intensive case management focusing prenatal care on prematurity prevention, early detection of preterm labor, and effective acute tocolysis, as well as reduced activity at home after tocolysis can complement maintenance tocolysis as described above in prolonging pregnancies and allowing better neonatal outcomes. 

## Figures and Tables

**Table 1:** Evidence-based medicine and continuous subcutaneous terbutaline infusion review of efficacy literature.

Study	Research design	Quality of evidence	*N*	Population	Comments
Lam et al. [11]	Observational cohort	II-2	1,556	RPTL	Inclusion criterion: tocolytic breakthrough in high-risk population subset. Results: PTD rate was reduced from 5.18% to 2.69% (*P* < 0.05).

Lam et al. [12]	Descriptive case series	III	9	RPTL	Pregnancy prolongation 9.2 weeks, mean GAD 39 weeks.

Lam et al. [13]	Randomized controlled trial (RCT)	I	68	CPTL	Weeks of pregnancy prolongation and PPI were 8.6 (0.93) and 2.4 (0.34) in the pump versus oral groups, respectively.

Gianopoulos et al. [14]	Descriptive case series	III	31	RPTL	Pregnancy prolongation 5.4 ± 4.5 weeks and 34.2 ± 3.8 weeks gestational age at delivery.

Jones et al. [15]	Descriptive case series	III	50	RPTL	Pregnancy prolongation 6.3 weeks.

Fischer and Kaatz [16]	Descriptive case series	III	19	CPTL	Safe and effective in the treatment of preterm labor. Average GAD 35.6 weeks.

McGettigan et al. [17]	Observational cohort	II-2	28	RPTL	Average GAD 35.7 weeks. Terb pump prolongs tocolysis, reduces terb dose significantly (*P* < 0.001), reduces maternal side effects, and may reduce the newborns' total exposure to *β*-mimetic dosage.

Wolfsen and Winn [18]	Descriptive case series	III	9	Twins w/advanced cervical dilatation	75% achieved >37 weeks or mature lung indices on amniocentesis

Allbert et al. [19]	Descriptive case series	III	992	C/RPTL 206 twins 786 singletons	Extended the gestation as mean of 38 ± 23 days and average GAD 36.3 ± 2.6 weeks.

Lindenbaum et al. [20]	Observational cohort	II-2	725	CPTL	The incidence of gestational diabetes is not increased in patients receiving terbutaline via the subcutaneous pump.

Moise et al. [21]	Descriptive case series	III	13	RPTL 10 singletons 2 triplets 1 twin	Average GAD 35.3 weeks, pregnancy prolongation 5.0 weeks.

Weinbaum and Olson [22]	Descriptive case series	III	202	CPTL	Contractions were arrested and the mean gestational age at delivery was 36.2 weeks. Only 9.6% of the patients were readmitted to the hospital.

Elliott and Radin [23]	Observational case control	II-2	67	CPTL 67 quadruplets	Mean GAD 32.5 weeks. Mean infant birth weight 1534 ± 429 g.

Adkins et al. [24]	Descriptive case series	III	51	CPTL	Average birth weight 3,000 g. Average GAD 37 weeks. Pregnancy prolongation 6.6 weeks.

Regenstein et al. [25]	Observational case control	II-2	151	CPTL	No difference in the incidence of gestational diabetes or glucose intolerance between subcutaneous and oral groups

Allbert et al. [26]	Observational cohort	II-2	64	RPTL	Pregnancy prolongation index was 0.86 and 0.72 for the pump and oral groups, respectively.

Perry et al. [27]	Descriptive case series	III	8,709	CPTL	Continuous terbutaline infusion is associated with much fewer adverse effects than the previously reported literature on intravenous terbutaline or ritodrine therapy would suggest.

Elliott et al. [28]	Retrospective cohort	II-2	21	CPTL 15 triplets 6 quadruplets	Estimated $18,150 savings per pregnancy. Only 2 of the 15 triplets (13%) and 1 of the 6 quadruplets (17%) delivered because of tocolytic failure. Mean GAD 33.0 weeks for both groups.

*Wenstrom et al. [29] *	*Randomized control trial (RCT) *	*I *	*42 *	*CPTL *	*Three-arm study—15 terbutaline pump, 15 oral terbutaline, and 12 saline pump. Significant methodological flaw in those patients crossed over between groups while in study. Patients on oral terbutaline or saline pump were switched to terbutaline pump if therapy failed. No electronic contraction monitoring or daily nursing contact. Tocolytic therapy was not individualized for each patient. Study underpowered in that it did not contain enough patients to show a difference between groups. No difference in outcomes between groups. *

*Guinn et al. [30] *	*Randomized control trial (RCT) *	*I *	*52 *	*CPTL *	*Overall dropout rate 38%. 13 patients in the terbutaline group completed this study and 19 patients in the placebo group. Advanced median cervical dilatation of 3 cm, effacement of 50% at start. Tocolytic therapy was not individualized for each patient. No electronic contraction monitoring or daily nursing contact. Study was underpowered and, therefore, showed no difference in outcomes between groups. *

Lam et al. [31]	Observational cohort	II-2	256	RPTL	Patients served as their own control. Subcutaneous terbutaline therapy prolonged pregnancy greater than oral terbutaline 4.4 ± 2.6 weeks compared to 2.7 ± 2.2 weeks.

Berkus et al. [32]	Descriptive case series	III	7	CPTL	Low dose, continuous SQ terbutaline infusion had no effect on insulin sensitivity in nondiabetic patients, in contrast to oral terbutaline.

Hammersley et al. [33]	Descriptive case series	III	70	RPTL 52 singletons 11 twins 7 triplets	Inclusion criterion: preterm labor or cervical shortening <3 cm and/or 50% funneling. Mean cervical length 2.6 ± 0.9 cm at initiation of therapy. 76% of desired pregnancy prolongation was achieved.

Lam et al. [34]	Observational cohort	II-2	386	RPTL 386 twins	34.0 ± 19.8 versus 19.3 ± 15.3 days in utero gained with subcutaneous therapy compared to oral therapy.

Ambrose et al. [35]	Observational case control	II-2	180	CPTL 76 twins	Outpatient-administered subcutaneous terbutaline shown to be a cost-effective and viable alternative versus inpatient-administered subcutaneous terbutaline.

Elliott et al. [36]	Observational case control	II-2	144	144 quadruplets	Outpatient therapy cost $30,270 less per patient and is associated with a statistically significant better chance of delivery >32 weeks than in patient.

Elliott et al. [37]	Observational cohort	II-2	104	RPTL 104 triplets	Mean pregnancy prolongation on pump 5.4 ± 3.4 weeks versus 2.8 ± 2.2 weeks for oral treatment.

*Lam et al. [38] *	*Observational case control *	*II-2 *	*706 *	*RPTL 706 twins *	*Total maternal and nursery charges were $17,109 less for patients treated with subcutaneous terbutaline compared to oral treatment. *

Elliott et al. [39]	Descriptive case series	III	9,359	CPTL 7,028 singletons 1,946 twins 385 triplets	Extremely low incidence of serious adverse events. GAD was 36.6 weeks in the singletons, 34.9 weeks in the twins, and 32.8 weeks in the triplets. Authors conclude that therapy is a viable and safe option for outpatient management.

Hamersley et al. [40]	Descriptive case series	III	6	Twins with delayed-interval delivery	The median pregnancy prolongation achieved following delivery of the first-born nonviable twin was 93 days.

Viscarello et al. [41]	Observational case control	II-2	40	CPTL 40 triplets	Proactive dose acceleration protocol achieved significantly better outcomes than standard dosing.

Viscarello et al. [42]	Observational case control	II-2	59	Higher order multiples: 56 triplets 3 quadruplets	A comprehensive clinical pathway (CCP) including subcutaneous terbutaline proved significantly better outcomes (35.1 ± 1.6 versus 31.6 ± 3.1 weeks GAD) compared to concurrent local standard of care. Of the 12 patients whose GAD was <32 weeks, 1 received the CCP including subcutaneous terbutaline, compared to 11 who received the concurrent local standard of care.

Jones et al. [43]	Descriptive case series	III	1420	CPTL	One-third of singletons and 60% of twins delivered within 3 days of early discontinuation of SQT. Early discontinuation of SQT places a pregnancy at risk for PTD.

*Morrison et al. [44] *	*Observational case control *	*II-2 *	*60 *	*RPTL *	*Among patients with recurrent PTL, the use of SQT infusion significantly prolongs pregnancy while decreasing the likelihood of the rate of low birth weight (2500 g) infants, the need for admission to NICU, and duration of being hospitalized. For every dollar spent on SQT, there was a saving of $4.67 on the charges of newborns stay in the hospital. *

*Lam et al. [45] *	*Observational case control *	*II-2 *	*558 *	*RPTL *	*70.6% of subcutaneous therapy patients reached at least 36 weeks compared to 56.6% of oral therapy patients. Subcutaneous therapy patients cost $5,286 less. *

Roman et al. [46]	Matched cohort	II-2	260	Twins in CPTL	>7 days prolongation of pregnancy in over 86% of cases

Gaziano et al. [47]	Matched cohort	II-2	1,079	CPTL	Outpatients obtained statistically better antepartum days, pregnancy prolongation, GAD, delivery <35 weeks, and cost. Total average cost outpatients were $17,375 versus $39,040 inpatient.

Rebarber et al. [48]	Matched cohort	II-2	783	CPTL	86% of patients had their pregnancy prolonged >7 days

*Fleming et al. [49] *	*Observational case control *	*II-2 *	*284 *	*RPTL *	*37.3% of nifedipine patients delivered ≤35 weeks compared to 19.7% of subcutaneous terbutaline patients. Subcutaneous terbutaline patients cost $10,494 less per pregnancy. *

Gaziano et al. [50]	Observational case control	II-2	273	CPTL	Twin pregnancies discharged for outpatient management following i.v. treatment for PTL obtained significantly longer pregnancy prolongation, a greater gestational age at delivery, and delivered infants with fewer NICU admission.

Brown and Stanziano [51]	Observational case control	II-2	840	CPTL	Medicaid versus commercial patients with singleton gestations and cervical dilatation of ≥2 cm at PTL. Medicaid patients experienced comparable pregnancy prolongation and gestational age at delivery as commercially insured. 96% of medicaid patients experienced pregnancy prolongation of at least 7 days after PTL. Incidence of discontinuation of SQT for noncompliance was 1.6% for medicaid versus 2.8% for commercially insured (*P* = 1.000).

Newman et al. [52]	Observational case control	II-2	1839	CPTL	Twin pregnancies with PTL. Medicaid versus commercially insured. Similar pregnancy prolongation and GA at delivery. 97% of medicaid patients experienced >7 days of pregnancy prolongation after PTL. Incidence of discontinuation of SQT for noncompliance was 4.6% for medicaid versus 2.0% for commercially insured (*P* = 0.076).

McWeeney et al. [53]	Descriptive	III	3496	CPTL	Singleton gestations hospitalized with CPTL; all treated with SQT following stabilization. The degree of cervical dilatation and gestational age at initiation of treatment are predictive of subsequent pregnancy outcome. With each centimeter of cervical dilatation, the risk for delivery at <32 weeks almost doubles.

Rebarber et al. [54]	Observational cohort	II-2	4253	CPTL	Singleton gestations with elective discontinuation of tocolytic treatment that occurred at 33–36 weeks' gestation were found to have a higher incidence of late preterm birth, with significantly greater rates of NICU admission and low birth weight, and significantly higher nursery charges. Tocolytic treatment should be continued through 36 weeks.

*de la Torre et al. [55] *	*Observational cohort *	*II-2 *	*1421 *	*Twins with RPTL *	*In twin pregnancies receiving nifedipine tocolysis, alteration of tocolytic treatment to subcutaneous terbutaline following hospitalization for recurrent preterm labor symptoms had a positive impact on pregnancy prolongation and neonatal outcomes. *

*Flick et al. [56] *	*Observational cohort *	*II-2 *	*4748 *	*Singletons with RPTL *	*Alteration of tocolytic treatment following rehospitalization for PTL resulted in decreased antepartum hospital days, decreased nursery days and lower rates of higher level nursery admission and preterm birth, proving both to be clinical and cost effective. *
